# Analysis of factors influencing the awareness of inpatients regarding hospital clinical pharmacy services and willingness to pay: a multicenter survey in Hebei Province, China

**DOI:** 10.3389/fphar.2024.1520058

**Published:** 2025-01-07

**Authors:** Ruixia Yang, Xin Wang, Huizhen Wu, Qian Sun, Sijie Zhang, Qian Dong, Mengjiao Li, Xiaoli Xu, Jing Bai, Ping Liang, Juan Hou

**Affiliations:** ^1^ Department of Pharmacy, The Fourth Hospital of Hebei Medical University, Shijiazhuang, China; ^2^ Department of Pharmacy, Hebei General Hospital, Shijiazhuang, China; ^3^ Department of Pharmacy, The Second Hospital of Hebei Medical University, Shijiazhuang, China; ^4^ Department of Cardiology, The Fourth Hospital of Hebei Medical University, Shijiazhuang, China; ^5^ Department of Oncology, The Fourth Hospital of Hebei Medical University, Shijiazhuang, China; ^6^ Medical Record Room, The Fourth Hospital of Hebei Medical University, Shijiazhuang, China

**Keywords:** pharmacy services, willingness to pay, inpatient awareness, clinical pharmacists, healthcare policy

## Abstract

**Introduction:**

Pharmacists are increasingly adopting patient-centered roles, improving healthcare outcomes by reducing medication errors and costs. In China, recent healthcare reforms recognize and compensate for pharmacy services. However, patient awareness of these services and their willingness to pay (WTP) remain underexplored. Therefore, this study aims to examine inpatient understanding of pharmacy services, their WTP in Hebei Province, and the factors affecting it.

**Methods:**

Between July and August 2024, a questionnaire was used to survey inpatients from 22 medical institutions across 11 prefecture-level cities in Hebei Province regarding their awareness of WTP for pharmacy services. Further investigation targeted inpatients unwilling to pay. The survey results were analyzed descriptively, with frequencies and percentages (%) used for categorical data and continuous data were presented as mean ± standard deviation (X ± SD). The chi-square test was used to determine statistically significant influences, and logistic regression analysis was performed to identify significant factors affecting inpatient WTP for pharmacy services. A column-line graph was validated using receiver operating characteristic curves and calibration graph analysis.

**Results:**

In total, 464 questionnaires were distributed, with 432 valid responses, yielding a 93.10% effective response rate. Most inpatients (89.58%) viewed hospital pharmacists as primarily responsible for dispensing medication, while only 5.79% were aware of broader pharmacy services. Despite this, 72.69% of the inpatients were willing to receive pharmacy services, and 95.38% of those who had received such services found them beneficial. Half of the inpatients (216, or 50.00%) indicated WTP for pharmacy services. Among those initially unwilling to pay (216 inpatients), 102 indicated they would consider payment if a doctor recommended clinical pharmacist guidance. Of the 318 inpatients who were WTP, 315 (99.06%) chose health insurance reimbursement as a payment method. Key factors influencing inpatient WTP included literacy level, preferred source of medication counseling, prior pharmacy service experience, understanding of pharmacy service policies, and readiness to recommend these services (*P* < 0.05).

**Conclusion:**

Most inpatients lacked knowledge and trust in clinical pharmacists, with limited awareness of the value of pharmacy services. However, they demonstrated high acceptance and WTP for these services, with nearly all inpatients preferring health insurance reimbursement. Integrating pharmacy service fees into health insurance is crucial for promoting these services.

## 1 Introduction

Recently, hospital pharmacists have shifted their focus from drug dispensing to a patient-centered approach (Jaber et al., 2019). Clinical pharmacy is becoming increasingly central to hospital pharmacy, highlighting the growing prominence of clinical pharmacists. As accessible and trusted members of the healthcare team (Anderson et al., 2022), hospital pharmacists play key roles in clinical drug therapy, ensuring rational medication use while enhancing their professional recognition and value (Chen et al., 2023). A review of 18 studies (from the BMJ, JAMA, LANCET, and NEJM) highlights the crucial role of clinical pharmacists in improving treatment outcomes and patient quality of life (Li et al., 2024a). Guidance from clinical pharmacists reduces medication errors and prevents adverse drug events (Boşnak et al., 2019; Murray et al., 2009; Schnipper et al., 2006). Furthermore, clinical pharmacists significantly reduce healthcare costs by lowering readmission rates, shortening hospital stays, and minimizing medical errors and medication discrepancies (Soodi et al., 2023). Many studies highlight their role in promoting safe, effective, and cost-efficient medication use, contributing to positive patient experiences and increased public acceptance of pharmacy services (Jaber et al., 2019; Anderson et al., 2022). However, sustaining these benefits requires a fee-for-service (FFS) system to ensure the long-term viability of pharmacy services (Hussain and Babar, 2023).

Quantifying the economic value of pharmacy services is essential (Painter et al., 2018). The need to assess their cost-effectiveness was recognized in 1971, with cost-benefit analysis as a common evaluation method (American Pharmacists Association, 1971). Among these, willingness to pay (WTP) is particularly suited for evaluating unlisted services within a cost-benefit framework, representing the monetary benefits and costs involved (AlShayban et al., 2020). WTP is calculated using contingent valuation methods, reflecting the amount a patient is willing to pay for a specific service (AlShayban et al., 2020; Jackson et al., 2023; Soodi et al., 2023). A study shows that over 50% of patients are willing to pay for pharmacy services (Xie et al., 2024b), although most prefer these services to be free (Murry et al., 2023). Factors influencing WTP include income, literacy, type of health insurance, and reimbursement policies (Du et al., 2024; Xie et al., 2024b).

There is a general consensus within the pharmacy community globally that future hospital pharmacy practice should focus on professional services aimed at patient health, with pharmacy services as the core (Liu et al., 2023). China has established a framework to compensate pharmacy services, supporting their implementation. Recently, the National Health Commission of the People’s Republic of China issued several documents encouraging healthcare institutions to expand pharmacy services, ensure fair compensation, and recognize the contributions of pharmacists. In 2023, the Commission introduced the National Technical Specification for Healthcare Service Items (2023 Edition), marking the first national inclusion of pharmacy service charges, such as pharmacist outpatient consultation, prescription dispensing, and inpatient medication monitoring (Du et al., 2024). The Health Commission of Hebei Provincial and Hebei Provincial Healthcare Security Administration initiated a trial Charging Policy for Pharmacy Services, including outpatient and inpatient consultation fees (with additional charges for clinical pharmacy) and multidisciplinary joint diagnosis and treatment (with additional charges for clinical pharmacy). This pilot program will run for 1 year. Hebei Province has a well-developed healthcare system, including strong medical security, a thriving traditional Chinese medicine sector, and an extensive network of medical institutions. This pilot program offers an opportunity to assess inpatient WTP for pharmacy services, allowing clinical pharmacists to enhance service quality and better meet inpatient needs.

Hospitals in Hebei Province are transitioning to a new pharmacy service model, presenting an opportunity to expand and refine service charges. As primary recipients of pharmacy services, inpatient perceptions, attitudes, and evaluations of clinical pharmacists directly impact the growth of pharmacy services (AlShayban et al., 2020; Jackson et al., 2023; Painter et al., 2018; Soodi et al., 2023). Assessing inpatients’ perceptions, attitudes, and WTP for pharmacy services in Hebei Province, China, and the factors influencing WTP is crucial. Furthermore, developing a prediction model to analyze the factors shaping inpatient WTP will guide future improvements in pharmacy services. Multifactor logistic regression analysis, an effective method for testing binary dependent variables, helps identify influencing factors (Xie et al., 2024a). A column chart visually represents a logistic regression model, illustrating the influence of variables on its output. However, research on using column-line diagram-based models to predict the factors influencing inpatient WTP is limited. Therefore, this study aims to construct a column-line diagram based on multivariate logistic regression from an inpatient survey on WTP for pharmacy services and evaluate the factors influencing WTP.

## 2 Materials and methods

### 2.1 Study design and patient population

This cross-sectional study was conducted in 22 hospitals across 11 prefecture-level cities in Hebei Province between July and August 2024. The selected hospitals were influential in their cities, representing the highest standard of medical service and medical facilities, extensive experience in clinical pharmacy services, and a willingness to participate, ensuring the objectivity of the study compared to private hospitals with potential conflicts of interest or bias. The study focused on patients with chronic diseases who typically require long-term drug treatment and have significant experience with pharmacy services. A total of 48 clinical pharmacists from 22 hospitals, each with over 3 years of experience in clinical drug therapy, conducted face-to-face interviews with inpatients using a simple non-random sampling method (Xie et al., 2024b).

Eligibility criteria included informed consent, voluntary participation, and basic communication ability. Patients with language or communication disorders were excluded from the study.

### 2.2 Questionnaire information

A comprehensive search of the CNKI, PubMed, Embase, and Web of Science databases was conducted using the keywords “willingness to pay,” “pharmacist,” “service,” and “consultation” to identify relevant literature. The initial questionnaire was drafted by frontline pharmacy personnel familiar with clinical pharmacy, specifically clinical pharmacists involved in drug therapy, and refined based on expert feedback and pre-survey results. This final questionnaire included input from the pharmacy department head and the clinical department director. Before the formal survey, clinical pharmacists underwent standardized online training to review the questionnaire content and objectives.

The questionnaire consisted of four sections. The first section was designed to ascertain basic demographic information (sex, age, and educational level). The second section assessed inpatients’ perceptions and needs regarding the role of clinical pharmacists. The third section addressed their views and expectations regarding pharmacy services. The fourth section evaluated their WTP for these services. Using a contingent valuation approach, WTP and attitudes toward pharmacy services were assessed through closed-ended questions. Patients were asked to indicate “yes” or “no” on the questionnaire if they were willing to pay for pharmacy services, their preferred reimbursement method, and the amount they were willing to pay via an open-ended question: “How much should pharmacy services cost?” (1 RMB = 0.14 USD).” Based on the pre-survey results, patients indicated the maximum amount they would pay for services, guided by national/provincial fee-related regulations, physician registration fees, and specific values. If unwilling to pay for pharmacy services, they were asked to explain why. The closed-ended question: “Would you be willing to pay for additional pharmacy instruction from a clinical pharmacist when recommended by a physician?” was used to assess inpatient WTP for pharmacy services. See Supplementary Material for the full questionnaire.

### 2.3 Validity and reliability

Cronbach’s alpha coefficient was used to assess the reliability of the questionnaire, yielding a value of 0.707, confirming the reliability of the research data for further analysis. Validity was tested using the KMO and Bartlett’s spherical tests, showing a KMO value of 0.761, indicating a strong factor relationship, with Bartlett’s spherical test (*P* < 0.001) confirming good structural validity.

### 2.4 Sample size

A literature review shows that 77.64% of inpatients support charging for pharmacy services (Li et al., 2024b), with a *P*-value of 0.7764, a Z-statistic of 1.96 (95% confidence), and a margin of error of 4%. Using the sample size formula for cross-sectional research (Eldridge et al., 2006), the required sample size was calculated as 417. Accounting for an estimated nonresponse rate of 5%–10%, the final sample size was 463.

$$n=\frac{{\left(Z_{1-\alpha }\right)}^{2}P\left(1-P\right)}{e^{2}}$$

### 2.5 Data collection and processing

The questionnaires were distributed to the participating clinical pharmacists through online questionnaires, with completion data primarily collected online. For hospitals with restricted online access or limited experience with online surveys, paper questionnaires were available, and completed questionnaires were collected by courier or fax. Participation was voluntary, and all data were anonymized and stored in password-protected files accessible only to the study team to ensure confidentiality. No monetary or other incentives were offered to participants. Before participation, all respondents were provided with detailed information about the nature and purpose of the survey and they could opt out at any stage.

### 2.6 Data analyses

After importing the raw data into Excel, invalid data entries were removed, and logical errors were checked. The cleaned data were then analyzed using IBM SPSS 21, R 4.4.1, and Python 3.12.4. Descriptive statistics were used to outline patient demographics, and the results were expressed as frequencies and percentages (%). Continuous data were reported as mean with standard deviation and median with interquartile range. The chi-square (χ2) test was utilized to identify statistically significant associations, while logistic regression was used to identify key factors influencing patient WTP for pharmacy services. Column-line graphs were constructed based on these factors, with accuracy validated through cohort verification, receiver operating characteristic (ROC) curve analysis, and area under the curve (AUC) assessment. Calibration plots were used to further validate predictive accuracy. A two-tailed P-value of 0.05 was considered significant at the 95% confidence interval.

### 2.7 Ethics approval and consent

This study was approved by the Medical Ethics Committee of the Fourth Hospital of Hebei Medical University (approval number 2023KS281). Only consenting inpatients, informed of the study objectives, were included in the data collection.

## 3 Results

In total, 464 questionnaires were received from 20 Grade-A tertiary and 2 Grade-A secondary hospitals across 11 prefecture-level cities in Hebei Province. Of these, 432 questionnaires were fully completed, yielding a valid response rate of approximately 93.10%. The respondents were primarily in the specialties of cardiovascular medicine, respiratory medicine, neurology, and oncology.

### 3.1 Basic information about inpatients

**FIGURE 1:**
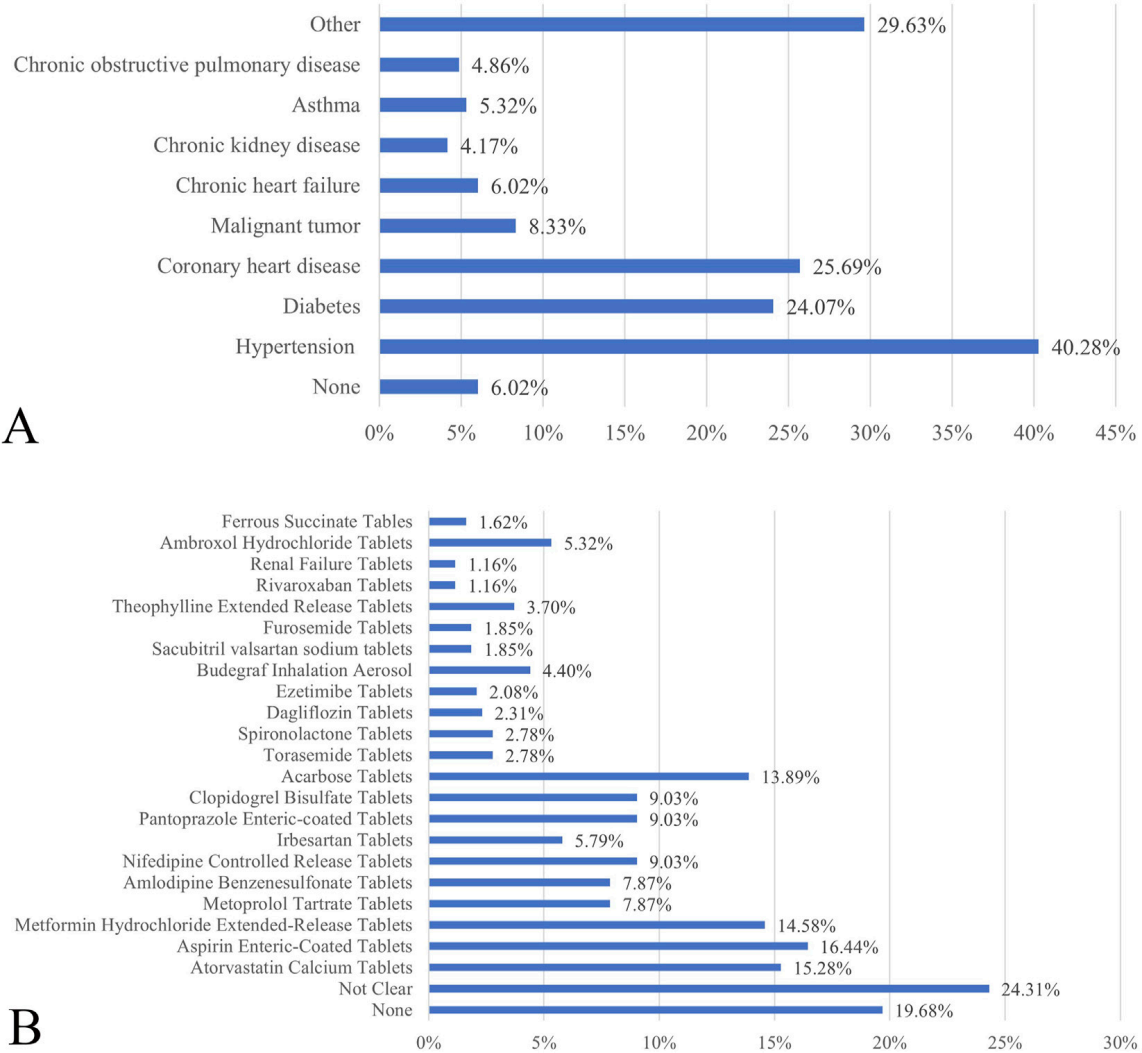
**(A)** The disease suffered by inpatients. **(B)** The medication taken by inpatients.

The survey revealed that 56.94% of the respondents were male, with the majority aged 35–59 (41.20%) and 60–74 (34.72%) years. The average age was 55.81 ± 16.08 years, ranging from 14 to 90 years. Most respondents (72.45%) had completed high school or less, indicating a lower educational level. A significant percentage (78.24%) had one or more chronic conditions, with hypertension being the most common (40.28%), followed by coronary heart disease (25.69%) and diabetes mellitus (24.07%). Additionally, 80.32% of respondents were on at least one medication, with 62.73% taking fewer than five types of medication, and the highest number being 18. Common medications included aspirin enteric-coated, atorvastatin calcium, metformin hydrochloride extended-release, and acarbose tablets (Table 1; Figure 1).

**TABLE 1:** Basic information on survey respondents (n = 432).

Characteristics	Frequency (%)	Mean (SD)/median (IQR)
Sex		
Male	246 (56.94)	
Female	186 (43.06)	
Age		55.81 ± 16.08
0–17 years	2 (0.46)	
18–34 years	52 (12.03)	
35–59 years	178 (41.20)	
60–74 years	150 (34.72)	
>75 years	50 (11.57)	
Literacy level		
Primary school and below	102 (23.61)	
High school and below	211 (48.84)	
University and above	119 (27.55)	
Current residence		
Town	226 (52.31)	
Rural	206 (47.69)	
Living situation		
Living with family	382 (88.43)	
Individual residence	50 (11.57)	
Does anyone close to you^®^ have a medical background?		
Yes	118 (27.31)	
No	314 (72.69)	
Combined chronic disease type		
0	94 (21.76)	
<3	283 (65.51)	
≥3	55 (12.73)	
Number of medications		
0	85 (19.68)	
<5	271 (62.73)	
5–10	68 (15.74)	
>10	8 (1.85)	

^®^ Anyone close to you: This refers to individuals with a close relationship with the respondent, such as family members, close friends, or significant others within their immediate social circle or household.

### 3.2 Perceptions and needs of inpatients for clinical pharmacists

**FIGURE 2:**
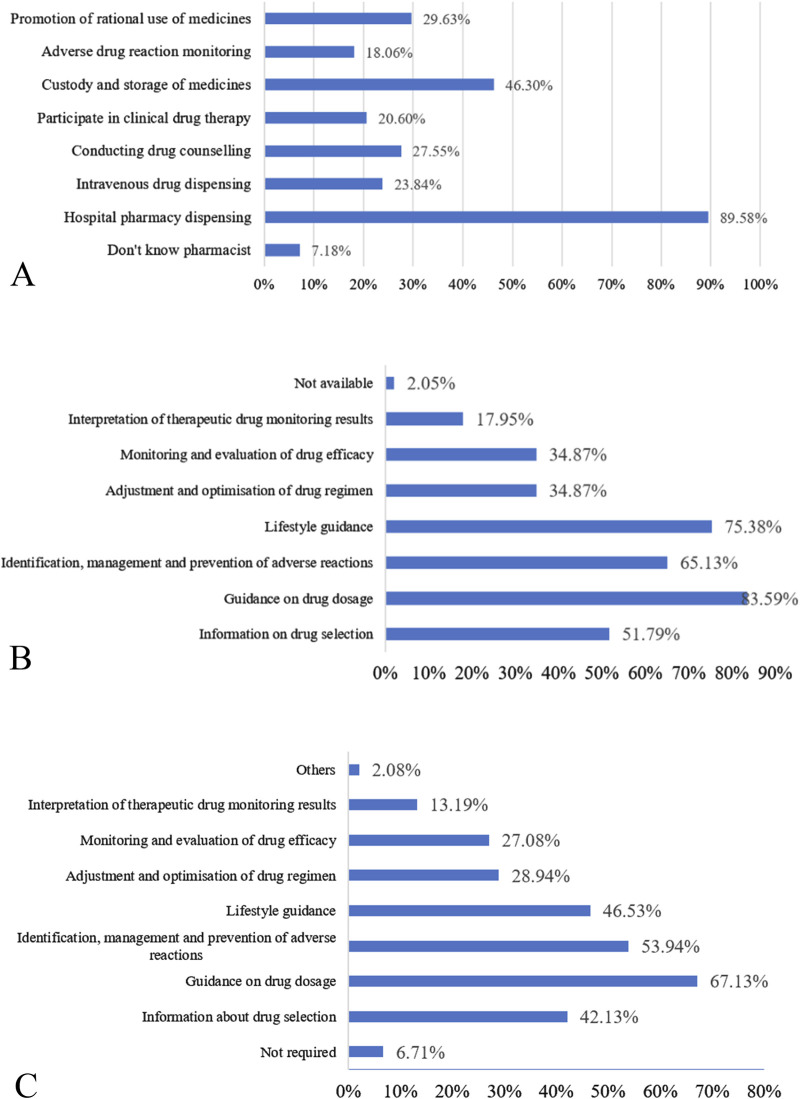
**(A)** Perceptions of inpatients regarding pharmacist roles. **(B)** Current exposure of inpatients to pharmacy services. **(C)** Demand of inpatients for pharmacy service programs.

The survey revealed that 89.58% of inpatients viewed pharmacists as responsible for dispensing medications. Conversely, fewer inpatients were aware of the involvement of pharmacists in drug counseling (27.55%), clinical drug therapy (20.60%), and promoting rational medicine use (29.63%). Furthermore, 7.18% of inpatients were unaware of the pharmacists’ roles and responsibilities (Figure 2A). Only 9.95% of inpatients considered consulting a pharmacist for medication-related issues, whereas 77.08% preferred seeking assistance from a medical practitioner. However, 69.98% of inpatients desired professional guidance from a clinical pharmacist during their hospital stay, although 10.42% felt they did not need the input of a clinical pharmacist during treatment. These results suggest that inpatients still associate pharmacists with traditional hospital pharmacy roles and trust doctors more than pharmacists.

### 3.3 Perceptions and needs of inpatients for pharmacy services

The survey showed that only 5.79% of inpatients knew the specifics of pharmacy services. While 32.64% of inpatients had heard of “pharmacy services,” they lacked detailed understanding. However, most inpatients were unaware of pharmacy services before the survey, with only 2.78% familiar with the pharmacy service fee policy, while 81.02% had never heard this information. This suggests limited awareness of the full scope of pharmacy services among inpatients.

In total, 45.14% of inpatients reported receiving pharmacy services during hospitalization, although 2.05% were unsure about the services they received. Among those who received pharmacy services, the most common were drug usage and dosage guidance (83.59%), lifestyle advice (75.38%), and the identification, treatment, and prevention of adverse reactions (65.13%) (Figure 2B). Most inpatients (95.38%) found the pharmacy services helpful in treating their illness, though 4.10% were unsure about their effectiveness, and 0.52% deemed them unhelpful. Additionally, 33.33% of inpatients identified gaps in aftercare and rehabilitation guidance provided by clinical pharmacists after their discharge. Furthermore, 13.85% of inpatients reported poor coordination between pharmacy professionals and medical doctors, leading to medication management discrepancies. Additionally, 8.21% of inpatients noted communication challenges with clinical pharmacists, while 5.74% stated that clinical pharmacists lacked relevant information on specific medicines and knowledge of proprietary Chinese medicines. Nevertheless, the vast majority of patients expressed a willingness to receive pharmacy services from clinical pharmacists (Table 2), indicating significant potential for improving pharmacy service delivery.

**TABLE 2:** Inpatient perceptions of pharmacy services.

Inpatients who did not receive pharmacy services (n = 237, 54.86%)		Inpatients who had received pharmacy services (n = 195, 45.14%)	
Item	Frequency (%)	Item	Frequency (%)
Do you think it is necessary for a clinical pharmacist to provide pharmacy services to you or your family members?		Do you think the pharmacy services provided by the clinical pharmacist are helpful to the treatment of your disease?	
Necessary	141 (59.49%)	Helpful	186 (95.38%)
Not sure	65 (27.43%)	Not sure	8 (4.10%)
It is not necessary	31 (13.08%)	Not helpful	1 (0.52%)
Are you willing to let a clinical pharmacist provide pharmacy services to you?		Are you willing to let a clinical pharmacist provide pharmacy services to you?	
Willing	147 (62.03%)	Willing	167 (85.64%)
Depends on the circumstances	71 (29.96%)	Depends on the circumstances	28 (14.36%)
Unwilling	19 (8.02%)	Unwilling	0
If a clinical pharmacist provides pharmacy services to you, which way would you prefer to communicate with the clinical pharmacist?		What do you think is the most important deficiency of the clinical pharmacist in providing pharmacy services to you or your family members?	
Face-to-face communication	203 (85.65%)	Not available	104 (53.33%)
Telephone communication	16 (6.75%)	Difficulty communicating with clinical pharmacists	16 (8.21%)
Online counseling	6 (2.53%)	Poor co-operation between clinical pharmacists and doctors	27 (13.85%)
Other	12 (5.06%)	Lack of relevant drug information by clinical pharmacist	11 (5.64%)
		Lack of discharge follow-up	65 (33.33%)
		Other	11 (5.64%)

Among inpatients who did not receive pharmacy services, 59.49% considered them necessary, while 27.43% were unsure. Nevertheless, 29.96% of inpatients expressed willingness to receive pharmacy services if a clinical pharmacist was needed during treatment. Most inpatients preferred face-to-face communication with clinical pharmacists (Table 2), indicating high acceptance of these services.

The most preferred pharmacy services were guidance on drug usage and dosage (67.13%), followed by identification, treatment, and prevention of adverse reactions (53.94%) and lifestyle advice (46.53%) (Figure 2C). Only 6.71% of the inpatients felt they did not need a clinical pharmacist. Additionally, 59.26% of inpatients indicated that they would recommend clinical pharmacists and pharmacy services to others if they faced medication-related issues, whereas 6.02% were reluctant to do so.

### 3.4 Willingness of inpatients to pay for pharmacy services

The survey showed that 216 inpatients (50.00%) indicated their WTP for pharmacy services. In a follow-up, 102 inpatients who were initially unwilling to pay changed their stance after the doctor offered additional pharmacy guidance from the clinical pharmacist. Reasons for reluctance to pay for these services were also explored, with 57.78% of inpatients believing that doctors could adequately address their medication-related questions, 33.33% viewing pharmacy services as a free professional responsibility, and 6.48% questioning the competence of the clinical pharmacist.

Of the 318 inpatients who expressed WTP, 315 (99.06%) chose the health insurance reimbursement option. Regarding pricing, 73.90% of respondents indicated that they would pay based on the relevant health insurance guidelines, while 20.13% indicated that they would refer to the registration fee of the physician. Additionally, 19 patients (5.97%) proposed specific fees ranging from $0.14 to $7.02, with an average of $1.77 and a median of $1.40.

### 3.5 Analysis of factors influencing the willingness of inpatients to pay for pharmacy services

#### 3.5.1 Differential analysis of the willingness of inpatients to pay for pharmacy services

The dependent variable was inpatients WTP for pharmacy services (whether or not they were willing to pay). Sixteen factors potentially influencing WTP were examined as independent variables. The chi-square test was employed to evaluate differences in inpatients’ WTP based on these factors, with statistical significance set at *P* < 0.05. The results showed that several factors significantly influenced inpatients’ WTP for pharmacy services (Table 3). These factors included cultural level, current residence, proximity to individuals with medical backgrounds, preferred source for medication counseling, need for clinical pharmacist assistance, awareness and prior receipt of pharmacy services, perceived necessity of pharmacy services, willingness to accept pharmacy services, familiarity with pharmacy service policies, and willingness to recommend pharmacy services to others. The results showed a statistically significant difference (*P* < 0.05).

**TABLE 3:** Differential analysis of the willingness of inpatients to pay for pharmacy services.

Factors	Inpatient willingness to pay for pharmacy services		X^2^	P
	Willing (n = 216)	Unwilling (n = 216)		
Sex			0.34	0.56
Male	126	120		
Female	90	96		
Age			8.755	0.068
0–17 years	1	1		
18–34 years	29	23		
35–59 years	101	77		
60–74 years	62	88		
>75 years	23	27		
Literacy level			31.447	<0.001
Primary school and below	38	64		
High school and below	93	118		
University and above	85	34		
Current residence			14.947	<0.001
Town	133	93		
Rural	83	123		
Living situation			2.262	0.133
Living with family	186	196		
Individual residence	30	20		
Does anyone close to you have a medical background?			16.836	<0.001
Yes	78	40		
No	138	176		
Combined chronic disease type			0.21	0.9
0	46	48		
<3	141	142		
≥3	29	26		
Number of medications			7.455	0.059
0	35	50		
<5	149	122		
5–10	28	40		
>10	4	4		
Preferred recipients of medication counseling			16.997	0.001
The Internet and Drug Formulary	14	20		
Clinical pharmacists	34	9		
Nurses	9	13		
Doctors	159	174		
Need help from clinical pharmacist assistance			43.886	<0.001
Need	182	119		
Does not matter	24	62		
No need	10	35		
Knowledge of pharmacy services			35.221	<0.001
Yes, I know	17	8		
Heard of it	96	45		
Heard of it for the first time	103	163		
Has the clinical pharmacist ever provided pharmacy services?			49.813	<0.001
Yes	134	61		
No	82	155		
Is pharmacy service necessary?			62.087	<0.001
Necessary	198	129		
Not sure	16	57		
Not necessary	2	30		
Willingness to receive pharmacy services			41.431	<0.001
Willing	185	130		
Depends on the circumstances	31	68		
Not willing	0	18		
Knowledge of pharmacy services policy			43.893	<0.001
Understand and aware	10	2		
Heard of it	58	12		
Heard of it for the first time	148	202		
Willing to recommend to others			60.227	<0.001
Willing to	167	89		
Depends on the situation	45	105		
Not willing	4	22		

#### 3.5.2 Multifactorial logistic regression analysis of factors influencing inpatient WTP

The dependent variable (Y) was inpatient WTP for pharmacy services, while the independent variables (X) consisted of 11 factors identified as statistically significant (*P* < 0.05) in previous univariate analyses. Binary logistic regression analyses were then performed using these variables. Literacy level, preferred source of medication counseling, prior receipt of pharmacy services, awareness of pharmacy service policies, and willingness to recommend pharmacy services to others influenced inpatients’ WTP for pharmacy services (Table 4, *P* < 0.05).

**TABLE 4:** Binary logistic regression analysis of factors influencing inpatient willingness to pay for pharmacy services.

Variables	B	SE	Wald	df	P	OR	95% CI	
							Lower	Upper
X_1_ Literacy level			8.37	2	0.015			
Primary school and below^®^								
High school and below	−0.261	0.31	0.709	1	0.4	0.77	0.42	1.414
University and above	−1.116	0.412	7.334	1	0.007	0.328	0.146	0.735
X_2_ Current residence								
Town^®^								
Rural	0.287	0.276	1.081	1	0.298	1.332	0.776	2.287
X_3_ Does anyone close to you have a medical background?								
Yes^®^								
No	0.263	0.303	0.751	1	0.386	1.301	0.718	2.357
X_4_ Preferred recipients of medication counseling			7.986	3	0.046			
The Internet and Drug Formulary^®^								
Clinical pharmacists	−1.541	0.654	5.556	1	0.018	0.214	0.06	0.771
Nurses	−1.302	0.71	3.36	1	0.067	0.272	0.068	1.094
Doctors	−1.304	0.481	7.352	1	0.007	0.271	0.106	0.697
X_5_ Need help from clinical pharmacists			2.277	2	0.32			
Need^®^								
Does not matter	0.511	0.34	2.26	1	0.133	1.666	0.856	3.242
No need	0.217	0.535	0.165	1	0.685	1.243	0.435	3.548
X_6_ Knowledge of pharmacy services			2.936	2	0.23			
Yes, I know^®^								
Heard of it	−0.444	0.654	0.46	1	0.498	0.642	0.178	2.313
Heard of it for the first time	0.029	0.651	0.002	1	0.965	1.029	0.288	3.683
X_7_ Has the clinical pharmacist ever provided pharmacy services?								
Yes^®^								
No	0.946	0.256	13.642	1	0	2.574	1.559	4.252
X_8_ Is pharmacy service necessary?			3.864	2	0.145			
Necessary^®^								
Not sure	0.605	0.405	2.228	1	0.136	1.831	0.827	4.053
Not necessary	1.346	0.855	2.477	1	0.116	3.84	0.719	20.521
X_9_ Willingness to receive pharmacy services			0.687	2	0.709			
Willing^®^								
Depends on the circumstances	0.286	0.345	0.687	1	0.407	1.331	0.677	2.615
Not willing	19.203	8893.127	0	1	0.998	2.19E+08	0	
X_10_ Knowledge of pharmacy services policy			10.993	2	0.004			
Understand and aware^®^								
Heard of it	−0.325	1.008	0.104	1	0.747	0.723	0.1	5.214
Heard of it for the first time	1.03	0.948	1.179	1	0.278	2.801	0.436	17.969
X_11_ Willing to recommend to others			14.726	2	0.001			
Willing to^®^								
Depends on the situation	0.936	0.277	11.448	1	0.001	2.551	1.483	4.387
Not willing	1.536	0.678	5.136	1	0.023	4.644	1.231	17.527

^®^, reference case; CI, confidence interval.

### 3.6 Construction and validation of the nomogram

**FIGURE 3:**
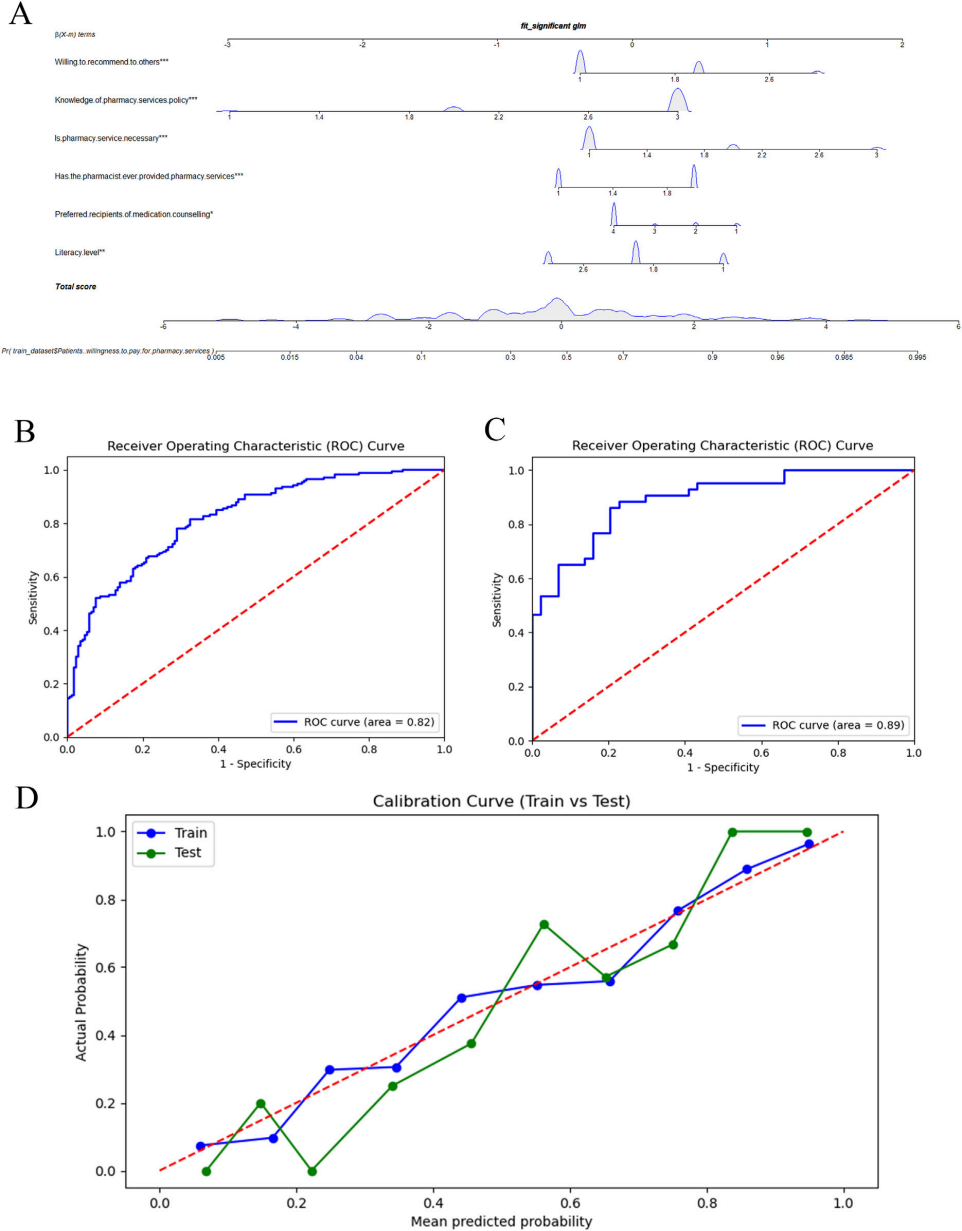
Nomogram to predict WTP. WTP, willingness to pay **(A)**. ROC curves for the predictive model in the training **(B)** and validation **(C)** cohorts. Calibration curve for predicting medication risks in the training (Train) and validation (Test) **(D)** cohorts.

A column-line diagram (Figure 3A) was initially constructed using multifactor logistic regression analysis to predict WTP. Key factors included inpatient awareness of pharmacy services, prior use of pharmacy services, and willingness to recommend pharmacy services to others. The results showed that inpatients with a university education or higher who sought advice from a medical professional for medication-related issues had previously used pharmacy services, were aware of pharmacy service pricing, and were inclined to recommend pharmacy services to others were more likely to be willing to pay. Furthermore, the predictive performance of the model was evaluated using ROC and calibration curves. Figures 3B, C show AUC values of 0.82 and 0.89 for the training and validation cohorts, respectively, indicating strong discriminative ability. Additionally, the calibration curve (Figure 3D) demonstrated good agreement between predictions and actual outcomes, supporting the predictive accuracy and reliability of the model. Overall, these findings suggest that the model is a highly accurate and effective tool for predicting inpatient WTP.

## 4 Discussion

WHO and the International Pharmaceutical Federation defines the goal of quality pharmacy education as producing “eight-star pharmacists”—health caretakers, decision makers, communicators, pharmacy leaders, managers, researchers, lifelong learners, and pharmacy trainers (Liu et al., 2023). In China, clinical pharmacists contribute significantly by educating patients, formulating medical orders, promoting personalized treatment, supervising problematic prescriptions, reporting adverse drug reactions, and disseminating pharmaceutical knowledge (Liu et al., 2023). Community pharmacists primarily focus on public health services and patient education in a few economically developed cities. Community pharmacy services remain underdeveloped compared to those of public medical institutions (Hu et al., 2024), where pharmacy services have significantly improved over time. Despite years of clinical pharmacy practice in Hebei Province, China, few inpatients remain unaware of the roles and responsibilities of pharmacists, primarily viewing them as drug dispensers, with only 5.79% (25/432) having a comprehensive understanding of pharmacy services. Awareness of pharmacy services in Hebei Province is significantly lower than that in Guizhou Province, China (29.11%, 68/233) (Li et al., 2024b). This may reflect Hebei’s ongoing transition from traditional hospital pharmacy practice to a developing “patient-centered” clinical pharmacy model (Li et al., 2023; Zhang et al., 2020). Enhancing pharmacy services and increasing public awareness of these services is essential. Despite the limited knowledge of inpatients regarding clinical pharmacists and low awareness of pharmacy services, most inpatients (69.98%) expressed a need for professional guidance from clinical pharmacists during treatment, willingness to accept pharmacy services (72.69%), and 75.69% considered these services beneficial (75.69%). These findings highlight the high demand for and acceptance of pharmacy care, indicating a promising future for its development. Therefore, clinical pharmacists should leverage new media platforms to share educational content, provide medication counseling, and engage in outreach efforts to gradually shift inpatient perceptions and enhance awareness of pharmacy services.

Most inpatients acknowledged the importance of pharmacy services in clinical care, with 77.08% (333/432) preferring to consult doctors for medication-related issues, while only a few sought help from clinical pharmacists. This aligns with findings from Guizhou Province, China (56.12%, 131/233) (Li et al., 2024b) and other published surveys (Chen et al., 2017; Mei et al., 2022). This preference reflects the long-established “doctor-led” healthcare approach in China and the slow development of clinical pharmacy (Li et al., 2023). Notably, 23.61% (102/432) of inpatients indicated that they would only consider paying for pharmacy consultations if recommended by their doctor, highlighting greater trust in doctors. However, owing to the heavy workload of doctors, many medication-related issues remain unresolved. With the rising prevalence of chronic diseases among Chinese adults and the need for long-term medication management, particularly for inpatients with chronic diseases, demand for pharmacy services, such as drug use and dosage guidance, adverse reaction management, and lifestyle counseling, is growing. Despite this increasing demand, clinical pharmacists must further enhance their expertise. Feedback from inpatients who received pharmacy services highlights significant areas for improvement, including updating clinical knowledge, strengthening communication with physicians, improving clinical reasoning, providing personalized care for inpatients, and building public trust in their role.

To our knowledge, this is the first provincial-level study in China to evaluate WTP for pharmacy services and its influencing factors and the second to assess inpatient WTP for pharmacy services after introducing the national FFS healthcare program. In this study, 73.61% (318/432) of inpatients expressed a WTP for pharmacy services. This result differs significantly from studies conducted in Guizhou Province, China (77. 64%, 181/233) (Li et al., 2024b), Brazil (93.25%, 525/563) (Tôrres et al., 2024), the United States (0%, 0/454) (Murry et al., 2023), Nigeria (45.08%, 182/403) (Nduka et al., 2023), and Saudi Arabia (29%, 154/531) (AlShayban et al., 2020). This contrast is particularly pronounced with findings from developed countries (United States, United Kingdom, Australia, Saudi Arabia, Switzerland, and South Korea) where WTP is significantly higher (Xie et al., 2024b). Studies from developing countries (China, Malaysia, Nigeria, Jordan, and Serbia) indicate that inpatients are generally less willing to pay, with an average WTP below $10. This variation likely stems from differences in pharmacy development stages, sample sizes, service scope, and pharmacy location. In the US, FFS pharmacy payment schemes vary by state, covering services such as medication therapy management (MTM), drug transfer fees, and medication counseling alongside value-based or hybrid performance-based fees (Hussain and Babar, 2023). In contrast, the UK’s simpler FFS model for pharmacy services, regulated by the National Health Service, compensates clinical pharmacists for professional services, with most services provided free to patients (Hussain and Babar, 2023). In Japan, pharmacy service fees depend on service complexity and required expertise, such as medication administration guidance, anesthetic support, and pharmacological monitoring, with fees varying based on patient risk (Du et al., 2024). In Alberta, Canada, pharmacy services fees are carefully categorized. For example, “medication review and assessment” is split into basic and advanced levels, with the average fee being approximately US$56 for 30 min. In Saudi Arabia, pharmacy service fees cover dispensing, MTM, and medication counseling (Hughes et al., 2017). In Nigeria, public health facilities typically offer free pharmacy services, while private facilities may charge a fee (Hussain and Babar, 2023). Similarly, in China, pharmacy service fees vary by province and cover outpatient and inpatient consultations (including clinical pharmacy services), and multidisciplinary treatments. Fujian, Hunan, and Hubei provinces have the broadest fee ranges, with most using per-patient or per-bed day fees. For instance, 11 provinces set inpatient consultation fees ranging from US$1.50 to US$2.25 per day. In Hebei Province, the Medical Insurance Bureau has set the fee at US$2.10 per day, with a maximum of US$6.30 for inpatients with a hospital stay of ≤30 days. However, the Health Commission of Hebei Province report that while 30 medical institutions provide this service, only 12 charge for it. Overall, establishing a fair reimbursement system is crucial to sustain and expand pharmacy services globally.

Logistic regression analyses and column-line graphs revealed key factors influencing inpatient WTP, including literacy level, preferred sources of medication advice, whether clinical pharmacist services were provided, awareness of pharmacy service policies on fees, and willingness to recommend the clinical pharmacist to others. Additionally, studies report that age (Jaber et al., 2019; Naik-Panvelkar et al., 2012; Soodi et al., 2023; Tôrres et al., 2024), sex (Tôrres et al., 2024), occupation (Tôrres et al., 2024; Jackson et al., 2023), satisfaction (AlShayban et al., 2020), location of pharmacy services (AlShayban et al., 2020; Hong et al., 2011), medical background (Lakić et al., 2017), and frequency of contact with clinical pharmacists (Lakić et al., 2017) influence WTP. However, some studies report no significant relationship between factors such as sex, age, income, satisfaction, and WTP (Friedrich et al., 2010; Gheewala et al., 2018). Further research is needed to explore these factors, as results may be influenced by sample size and development level of pharmacy service in different countries. In this study, inpatients with a university education or higher were more willing to pay for pharmacy services [odds ratio (OR) = 0.328, *p* = 0.007], aligning with findings from Tôrres et al. (2024) and Lakić et al. (2017). This trend may be attributed to lower education levels, as less educated inpatients may have limited access to new information and be less receptive to new ideas. Targeted efforts are needed to promote pharmacy services among these groups. Inpatients who preferred consulting their physician over the internet or package leaflets were more willing to pay for pharmacy services (OR = 0.271, *p* = 0.007), reflecting a general lack of awareness about safe medication use and the limited time physicians have to provide adequate guidance. Therefore, these inpatients are ideal candidates for pharmacy services, which can provide the necessary support. WTP was higher among those who had received services from clinical pharmacists (OR = 2.574, *p* < 0.001), aligning with the findings from AlShayban et al. (2020). This suggests that most inpatients recognize the expertise of clinical pharmacists and believe their services deserve payment. Furthermore, inpatients willing to recommend pharmacy services were more likely to pay for them (OR = 4.644, *p* = 0.023). This suggests that satisfied inpatients were more likely to pay for these services. Awareness of pharmacy service pricing policies was another significant factor (*p* = 0.004); inpatients familiar with the pricing policy recognized the value of pharmacy services and were more willing to pay for them. Further analysis revealed that reluctance to pay stemmed from limited awareness of the roles of clinical pharmacists and lack of understanding of pharmacy services. This led to over-reliance on doctors for medication guidance and the mistaken belief that medication safety is solely the responsibility of doctors, while clinical pharmacists were seen only as medication dispensers. Some inpatients viewed pharmacy services as part of medical care, seeing them as the duty of the clinical pharmacist rather than a separate, chargeable service. Additionally, a lack of trust in clinical pharmacists due to inadequate communication with inpatients who receive fewer pharmacy services results in doubts about their professional competence and reluctance to pay. Therefore, clinical pharmacists should be more involved in the diagnosis and treatment process, provide professional guidance and high-quality services, expand pharmacy service scope, and increase public recognition of the value of pharmacy services.

The survey showed that nearly all inpatients preferred health insurance reimbursement for pharmacy services. Inpatients with private health insurance demonstrated a higher WTP than those with only public health insurance, following a study by Jaber et al. (2019). This is because private health insurance typically covers more services, reducing out-of-pocket expenses for inpatients. Similarly, Anosike et al. (2020) report that health insurance reimbursement policies impact inpatient WTP. This suggests that including pharmacy services in reimbursement policies may significantly influence coverage and payment. Since 2006, the Centers for Medicare and Medicaid Services have included MTM programs in the US public health insurance system. Inpatients receiving MTM services can be reimbursed through Medicare, Medicaid, or private health insurance on a contractual basis (Zheng et al., 2021). Currently, only Beijing and Hunan provinces reimburse inpatient consultation fees (including clinical pharmacy services) under category A of medical insurance. Other provinces, including Hebei, allow these fees as part of medical insurance but inpatients still have the option for out-of-pocket payment. In summary, health insurance reimbursement policies significantly influence inpatient WTP for pharmacy services. Increasing reimbursement levels and proportions is an effective strategy to enhance WTP, expand coverage, and improve access to pharmacy services. This is crucial for improving medication safety therapeutic outcomes, reducing the risk of adverse drug reactions, and enhancing inpatient quality of life. Additionally, it supports the growth of the service industry and promotes healthcare equity and sustainability.

This study had some limitations. First, it used non-random sampling with a limited sample size, and most surveyed medical institutions were Grade-A tertiary hospitals. Although these institutions are regionally representative, further multicenter, stratified research across the province is still necessary. Second, healthcare workers and pharmacists were not included; hence, future studies should involve these groups to better understand their attitudes toward pharmacy services and WTP. Currently, no analysis of pharmacy services and WTP exists in Hebei Province. However, the findings of the study offer partial insights into the development of hospital pharmacy services in Hebei Province and inform the advancement of pharmacy service programs both locally and nationally.

In conclusion, most inpatients have limited knowledge and trust in pharmacists, coupled with low recognition of the value of pharmacy services. However, they show higher acceptance and WTP for these services. Factors influencing inpatient WTP for pharmacy services include literacy, preferred source of medication counseling, prior receipt of pharmacy services, awareness of pharmacy service policies, and willingness to recommend pharmacy services. Most participants prefer health insurance reimbursement for pharmacy services. Currently, hospital pharmacies in Hebei Province—and across China—are exploring and implementing changes to pharmacy services. The development of a pharmacy service fee system is still in the exploratory phase. Whether these fees are covered by health insurance reimbursement is crucial for broader adoption of these services. Therefore, clinical pharmacists must improve their professionalism through theoretical and practical research, demonstrating the value of their work and providing evidence for including pharmacy service fees in health insurance reimbursement.

## Data Availability

The original contributions presented in the study are included in the article/Supplementary Material, further inquiries can be directed to the corresponding authors.

## Abbreviations

WTP, willingness to pay; MTM, medication therapy management; AUC, area under the curve; ROC, receiver operating characteristic; OR, odds ratio.
