# Supplementary material for: Analysis of factors influencing the awareness of inpatients regarding hospital clinical pharmacy services and willingness to pay: a multicenter survey in Hebei Province, China
Source: Front Pharmacol. 2025 Jan 7;15:1520058. doi: 10.3389/fphar.2024.1520058 (PMC11746059; doi:10.3389/fphar.2024.1520058)
Supplement: Supplementary file 1 [file DataSheet1.docx]

Supplementary Material

# Inpatient Pharmacy Service Survey Questionnaire

**I. Basic Patient Information**

1. What is your gender: □Male □Female

2. What is your age: _____ years old

3. What is your level of education:

□Primary school and below □High school and below □University and above

4. Current Residence: □Town □Rural

5. Living situation: □Living with family □Living alone

6. Does anyone close you have a medical background: □Yes □No

7. What chronic diseases do you currently have:

□Hypertension □Diabetes □Coronary heart disease

□Malignant tumor □Chronic heart failure □Chronic kidney disease

□Asthma □Chronic obstructive pulmonary disease □Other:

8. What kinds of medications are you currently taking:

□kinds (can specify) ______________

**II. Survey Content**

1. Are you aware that hospital pharmacists engage in the following activities?

□Hospital pharmacy dispensing □Intravenous medication preparation

□Conducting medication consultations □Participating in clinical drug therapy

□Drug storage and preservation □Monitoring of adverse drug reactions

□Promoting rational drug use knowledge □Other

2. When encountering medication-related issues, who do you first think of consulting?

□Doctor □Nurse □Clinical pharmacists □The Internet and Drug Formulary

3. When you or your family are hospitalized, do you need a clinical pharmacist to come to the bedside to care about medication use and provide professional guidance and assistance?

□Need □Doesn't matter □Don't need

4. Are you aware that medication monitoring, such as efficacy and adverse reaction monitoring during medication use, medication guidance, personalized medication advice, and blood drug concentration monitoring, all fall under the category of pharmacy services?

□Yes, I know □Heard of it □Heard of it for the first time

5. During your or your family's hospitalization or at discharge, has a clinical pharmacist provided you with the above pharmacy services?

□No:

①Do you think it is necessary for a clinical pharmacist to provide pharmacy services to you or your family members?

□Necessary □Not sure □It's not necessary

②Are you willing to accept pharmacy services provided by a pharmacist?

□Willing □Depends on the circumstances □Unwilling

③If a clinical pharmacist provides pharmacy services to you, which way would you prefer to communicate with the clinical pharmacist?

□Face-to-face communication □Telephone communication

□Online consultation □ Other

□Yes:

①What pharmacy services did the pharmacist provide you during hospitalization? (Multiple choices allowed)

□Information on drug selection □Guidance on drug usage and dosage

□Identification, treatment, and prevention of adverse reactions related to drugs

□Lifestyle guidance, health education

□Adjustment and optimization of drug treatment plans

□Monitoring and evaluation of drug efficacy

□Interpretation of therapeutic drug monitoring (such as blood drug concentration and pharmacogenetic testing) results □Other

②Do you think the pharmacy services provided by the clinical pharmacist are helpful to the treatment of your disease?

□Helpful □Not sure □Not helpful

③Are you willing to accept the clinical pharmacist to provide pharmacy service to you?

□Willing □Depends on the circumstances □Unwilling

④What do you think is the most important deficiency of the clinical pharmacist in providing pharmacy services to you or your family members?

□Not available

□Difficulty in communicating with the clinical pharmacist

□Poor co-operation between clinical pharmacists and doctors

□Lack of relevant drug information by clinical pharmacist

□Lack of discharge follow-up □Other

6. Which medication-related issues are you more willing to consult a pharmacist about?

□No need

□Information on drug selection □Guidance on drug usage and dosage

□Identification, treatment, and prevention of adverse reactions related to drugs

□Lifestyle guidance, health education

□Adjustment and optimization of drug treatment plans

□Monitoring and evaluation of drug efficacy

□Interpretation of therapeutic drug monitoring (such as blood drug concentration and pharmacogenetic testing) results □Other

7. Have you previously heard or learned about the Hebei Province's policy or news regarding the charging of pharmacy services?

□Heard of it for the first time □Heard of it □Understand and know

8. If pharmacy services require a fee, are you willing to accept pharmacy services provided by a pharmacist?

□Willing (only answer options ②③) □Unwilling

The reasons for your unwillingness to pay are:

□Prefer to consult a doctor □Think pharmacy services should be free

□Do not need pharmacy services during treatment

□Doubt the effectiveness of pharmacy services □ Other

①If a physician suggests further pharmacy guidance for you and your family, are you willing to accept pharmacy services and pay for it?

□Unwilling □Willing

②The way you are willing to pay for pharmacy services is:

□Health insurance payment □Out-of-pocket

③What do you think the price for pharmacy services should be?

□According to national/province fee regulations is fine

□Can refer to physician consultation fees

□_____ yuan (specific amount)

9. When others encounter medication-related issues, are you willing to recommend pharmacy services to others?

□Willing to □Depends on the situation □Not willing
